# Pediatric and Adult High-Grade Glioma Stem Cell Culture Models Are Permissive to Lytic Infection with Parvovirus H-1

**DOI:** 10.3390/v8050138

**Published:** 2016-05-19

**Authors:** Rafael Josupeit, Sebastian Bender, Sonja Kern, Barbara Leuchs, Thomas Hielscher, Christel Herold-Mende, Jörg R. Schlehofer, Christiane Dinsart, Olaf Witt, Jean Rommelaere, Jeannine Lacroix

**Affiliations:** 1 Research Program Infection, Inflammation and Cancer, Division of Tumor Virology, German Cancer Research Center (DKFZ), Im Neuenheimer Feld 242, 69120 Heidelberg, Germany; rafael.josupeit@googlemail.com (R.J.); sonja.kern@uni-wuerzburg.de (S.K.); b.leuchs@dkfz-heidelberg.de (B.L.); joteres@arcor.de (J.R.S.); c.dinsart@dkfz-heidelberg.de (C.D.); j.rommelaere@dkfz-heidelberg.de (J.R.); 2 Division of Pediatric Neurooncology, German Cancer Research Center (DKFZ), Im Neuenheimer Feld 580, 69120 Heidelberg, Germany; SeBender@gmx.de; 3 Division of Biostatistics, German Cancer Research Center (DKFZ), Im Neuenheimer Feld 580, 69120 Heidelberg, Germany; t.hielscher@dkfz.de; 4 Division of Experimental Neurosurgery, Department of Neurosurgery, University Hospital, Im Neuenheimer Feld 400, 69120 Heidelberg, Germany; h.mende@med.uni-heidelberg.de; 5 Clinical Cooperation Unit Pediatric Oncology, German Cancer Research Center (DKFZ), Im Neuenheimer Feld 280, 69120 Heidelberg, Germany; o.witt@dkfz.de; 6 Department of Pediatric Hematology, Oncology and Immunology, Center for Pediatric and Adolescent Medicine, University Hospital, Im Neuenheimer Feld 430, 69120 Heidelberg, Germany

**Keywords:** oncolytic virus, parvovirus H-1, H-1PV, DIPG, pediatric glioblastoma, tumor initiating cells, glioma stem-like cells, high-grade glioma, J0101

## Abstract

Combining virus-induced cytotoxic and immunotherapeutic effects, oncolytic virotherapy represents a promising therapeutic approach for high-grade glioma (HGG). A clinical trial has recently provided evidence for the clinical safety of the oncolytic parvovirus H-1 (H-1PV) in adult glioblastoma relapse patients. The present study assesses the efficacy of H-1PV in eliminating HGG initiating cells. H-1PV was able to enter and to transduce all HGG neurosphere culture models (*n* = 6), including cultures derived from adult glioblastoma, pediatric glioblastoma, and diffuse intrinsic pontine glioma. Cytotoxic effects induced by the virus have been observed in all HGG neurospheres at half maximal inhibitory concentration (IC50) doses of input virus between 1 and 10 plaque forming units per cell. H-1PV infection at this dose range was able to prevent tumorigenicity of NCH421k glioblastoma multiforme (GBM) “stem-like” cells in NOD/SCID mice. Interestingly NCH421R, an isogenic subclone with equal capacity of xenograft formation, but resistant to H-1PV infection could be isolated from the parental NCH421k culture. To reveal changes in gene expression associated with H-1PV resistance we performed a comparative gene expression analysis in these subclones. Several dysregulated genes encoding receptor proteins, endocytosis factors or regulators innate antiviral responses were identified and represent intriguing candidates for to further study molecular mechanisms of H-1PV resistance.

## 1. Introduction

High-grade glioma in children, adolescents, and adult patients comprises a spectrum of diseases, for which current treatment protocols only can offer little hope for cure. In contrast to common histopathological features, research on oncogenic pathways driving high-grade glioma formation revealed, that pediatric and adult high-grade glioma have to be considered as distinct disease entities, each of them comprising several molecular subgroups [1]. For primary glioblastoma multiforme (GBM), conventional treatment concepts include stereotactic biopsy or resection, radiotherapy, and adjuvant chemotherapy [2]. Resectability of the tumor has remained a main determinant of the outcome of the disease in pediatric high-grade glioma (HGG) patients for more than three decades [3,4]. In diffuse intrinsic pontine glioma patients (DIPG) prognosis is worst, since the procedural risk of surgical interventions excludes resections as a therapeutic option for these patients [5]. Since temozolomide had gained approval in 2005, no further treatment option has significantly improved survival of adult GBM patients. In children with HGG current clinical trials evaluate the therapeutic benefit of temozolomide treatment and the role of prognostic factors defined in adult GBM such as 1p19q status, isocitrate dehydrogenase (IDH) mutation status, and O^6^-methylguanine-DNA methyltransferase (MGMT) promoter methylation status [6].

During the last decade, an era of cancer research based on individualized therapies has emerged. The identification of druggable pathways is a pre-requisite for these targeted therapeutic approaches. Clinical trials for HGG patients provided first evidence of efficacy of targeted therapies, but showed that these are hampered by the high molecular heterogeneity of HGG tumors. Recruitment of patient groups of adequate size and homogeneity in molecular and clinical risk profiles remains a challenge [7]. The situation is further complicated by genetic and epi-genetic heterogeneity within a single tumor and by the heterogeneity observed in primary tumors compared to metastatic lesions [1,8,9]. HGG indeed comprises a variety of genetically and epi-genetically distinct cellular subtypes including subpopulations of cancer cells mimicking all states of neural and glial differentiation [10]. Therapeutic approaches targeting one or only some of these subpopulations are therefore unlikely to achieve sustained therapeutic responses. Oncolytic viruses, however, have the capacity to act as multi-target weapons against cancer. These viruses are able to exert direct cytotoxic effects by interacting with multiple cellular processes and by inducing or increasing anti-cancer immune responses.

In search of novel therapeutic approaches for HGG patients, oncolytic virotherapy has been pre-clinically evaluated in a variety of cell culture systems and animal models. Compared to other antineoplastic agents, self-replicating oncolytic viruses, including H-1 parvovirus (H-1PV), represent a unique treatment approach by combining cytotoxic effects with the induction of immune-mediated cancer cell death. These viruses are characterized by their ability to replicate in initially infected malignant cells, to induce successive rounds of productive infection and to establish thereby an effective virus dose. Moreover, infection of cancer cells with oncolytic viruses has been demonstrated to induce anti-tumoral immunity, shifting the immunological microenvironment within a tumor from tolerance to tumor antigens to a T helper cells type 1 (Th1) mediated anti-tumoral immune response [11]. The latter mode of action offers the unique advantage of sustained control of residual cancer cells even after elimination of the viral therapeutic agent.

In pre-clinical human glioblastoma models, a variety of oncolytic viruses have proven to induce significant cytotoxic effects *in vitro* and *in vivo* using orthotopic xenograft rodent models. These results have paved the way for clinical research in HGG patients, leading to an increasing number of early phase virotherapy trials [12]. In adult HGG patients, these first oncolytic virotherapy trials have provided evidence for the clinical safety of these therapeutic approaches and, to some extent, antineoplastic efficacy [13]. In particular, adult HGG has been shown to be a promising target for the application of the oncolytic protoparvovirus H-1PV. This self-replicating virus is endemic in rat populations. Its antineoplastic effects had been demonstrated *in vitro* and in both allograft and xenograft-bearing orthotopic rat models [14]. In the rat glioma allograft model long time survival has been observed after intratumoral, intravenous or intranasal virus application [15].

Based on these preclinical toxicity and safety data, a phase I/IIa clinical trial of H-1PV in adult patients with recurrent glioblastoma was launched in 2011 [16]. While clinical evaluation is still in progress, interesting information has been obtained regarding virus distribution, expression and effects on both tumor and immune cells. Furthermore, the trial has confirmed clinical safety after intratumoral and intravenous H-1PV administration [17].

HGG stem-like cell culture models and animal models derived thereof represent a new gold standard in pre-clinical testing of new anti-neoplastic agents. These models have been shown to recapitulate the distinctive cytological hallmarks and the histological variants associated with the initial tumor of the corresponding patients [18]. In adult glioma stem-like cells, cytotoxic effects have been reported *in vitro* for several oncolytic viruses including adenoviruses (AdV), [19], measles virus (MV) [20] and herpes simplex virus (HSV) [21]. In glioma stem cell derived xenotransplant models, significant suppression of glioma cell proliferation and improvement of survival was achieved using different types of genetically engineered oncolytic HSV [22,23] and MV derivatives [20]. Similar approaches remain to be evaluated in pediatric HGG stem cell models. First data on the administration of an oncolytic virus in pediatric HGG stem-cell cultures and animal models have been recently published [24], but data on antineoplastic efficacy are still lacking.

In the present study, we addressed the question, whether H-1PV is able to eradicate HGG stem cells. Neurosphere cultures derived from the most frequent HGG subtypes in adult (GBM) and pediatric (GBM and DIPG) patients served as *in vitro* models for pre-clinical testing. Pediatric HGG neurosphere culture models were characterized for the expression of the glioma stem cell markers CD133, Nestin and SOX-2, and compared to stem-like cells derived from adult glioblastoma previously described. The present study demonstrates for the first time, that H-1PV is able to induce lytic infection in HGG stem-like cells derived from adult and pediatric high-grade glioma, and to suppress tumorigenicity of glioma stem-like cell in SCID mice. This capacity represents an intrinsic property of H-1PV and does not require any modification of the wild type virus. Furthermore candidate cellular genes controlling viral entry and transduction in HGG-stem-like cells have been identified using this model.

## 2. Materials and Methods

### 2.1. Ethics Statement

The pediatric glioblastoma cell lines SF-188 and KNS-42 were obtained from the Department of Neurosurgery, University of California (San Francisco, CA, USA) and the Japan Health Science Research Resources Bank, (Osaka, Japan), respectively. The SF-188 NS and KNS-42 NS neurosphere subclones were generated by cultivating the parental lines under serum-free conditions as described above (“secondary neurospheres”). The neurosphere cultures SU-DIPG-IV, and SU-DIPG-VI, have been established from post mortem diffuse intrinsic pontine samples of two pediatric patients, and have been previously characterized [25,26]. These cultures were a kind gift of Michelle Monje-Deisseroth, University of Stanford (Stanford, CA, USA). The human glioma stem-like cell cultures NCH421k and NCH644 were derived from biopsies taken from adult glioblastoma patients and have been established the laboratory of Christel Herold-Mende. These cells had been previously reported to represent glioma “stem-like” cells [27].

Animal experiments were performed approved by the Institutional Animal Welfare Committee of the German Cancer Research Center, according to the German Animal Welfare Act (TierSchG) after approval by the state of Baden-Württemberg (permit numbers: 35-9185.81 G-3/12 and 35-9185.81 G-40/15 RP Karlsruhe, Germany).

### 2.2. Cell Culture

All cell lines were cultured at 37 °C in a 5% CO_2_ atmosphere. Adult GBM neurospheres (NCH 421k, NCH421R, NCH644) and pediatric GBM secondary neurospheres (SF-188NS and KNS-42NS) were cultured in Dulbecco's Modified Eagle Medium (DMEM) containing 20% BIT serum-free supplement, basic fibroblast growth factor (bFGF, 20 ng/mL), and epidermal growth factor (EGF, 20 ng/mL), (all from Provitro GmbH, Berlin, Germany), as previously described [27]. DIPG neurospheres (SU-DIPG IV, SU-DIPG VI) were cultured in a tumor stem medium consisting of Neurobasal-A (Invitrogen™, Life technologies GmbH, Darmstadt, Germany), B27-A (Invitrogen™), human bFGF, (20 ng/mL), human EGF (20 ng/mL), human platelet-derived growth factor-AB (PDGFab) (20 ng/mL) (all from Provitro) and heparin (10 ng/mL) as described [28].

All cell cultures were regularly monitored for the absence of pathogen contaminations, using the Multiplex cell Contamination Test (Multiplexion GmbH, Heidelberg, Germany) as published [29]. No *Mycoplasma* or squirrel monkey retrovirus (SMRV) or other non-human DNA contaminations were detected.

### 2.3. Generation of the Subclones and Cell Authentication

The NCH421R and NCH421I neurosphere cultures were spontaneously arising subclones isolated and characterized in our laboratory in course of the present project. Both are derived from the same, parental NCH421k neurosphere culture. Due to their significantly different response to H-1PV these subclones were initially thought to represent genetically different neurosphere cultures. However, the single nucleotide polymorphism (SNP) profiles of NCH421R, NCH421I and NCH421k showed perfect matching confirming their identical origin (see below). NCH421R is a subclone of NCH421k cells isolated in our lab for its resistance to repeated rounds of H-1PV infection. NCH421I is a third subclone derived from the parental NCH421k culture modeling limited susceptibility to H-1PV infection. High doses of input virus can transduce these cells, but only low levels of viral protein expression are detectable after infection with 50 plaque forming units (PFU) per cell. However, in contrast to the parental NCH421k cells, this subclone is completely deficient in virus replication.

Cell lines were authenticated using the Multiplex Cell Authentication test, and performed by a commercial service (Multiplexion GmbH, Heidelberg, Germany) [30]. The SNP profiles of all cell lines matched to published data or were unique. No interspecies contamination was detected.

### 2.4. Virus Production and Infection

H-1PV was produced by infection of NBK-324K human embryonic kidney cells, and purified by iodixanol (VisipaqueTM, Amersham Biosciences, Freiburg, Germany) gradient centrifugation and subsequent passage through a 0.2 μm filter. The contamination of virus stocks with endotoxins was lower than 2.5 EU/mL. Infection experiments were performed using appropriate serum-free cell culture medium.

### 2.5. Detection of Infectious H-1PV Particles by Propagation in NB-324k Cells

Virus titers were determined by endpoint dilution assay, after infection of cells and subsequent dot/blot detection of viral DNA. In order to quantify the increase of infectious progeny virions secreted by in HGG neurosphere cultures, time course experiments were performed. After H-1PV infection the cell culture medium of the neurosphere cultures were collected at the time points indicated. 24 h after being seeded in 96-well plates, NB-324K cells were infected with 10-fold serial dilutions of this suspension. The titer of H-1PV subsequently was determined by dot blot assay as described [28].

### 2.6. Flow Cytometric Analysis of CD133 Expression

For CD133 content analysis of the neurosphere cultures, cells were harvested, singularized and counted. A minimum of 1 × 10^7^ cells per sample were prepared. The analysis of each neurosphere culture necessitated five samples: (i) without fluorophore; (ii) 7-amino-actinomycin D (7AAD); (iii) CD133-PE sample for compensation purposes; (iv) IgG2b-phycoerythrin (PE) control mixed with 7AAD for discrimination of random antibody binding and (v) CD133-PE and 7AAD for the analysis presented. Staining was conducted according to the manufacturer’s instructions.

7AAD (Beckton Dickinson Biosciences, Heidelberg, Germany) was used as a cell viability stain to avoid false positive signals by dead cells. Analysis was performed on a fluorescence-activated cell sorting (FACS) Calibur instrument using the FlowJo software package (Tree Star Inc., Ashland, OR, USA). Briefly, cells were washed in phosphate-buffered saline (PBS) and subsequently resuspended in FACS buffer, containing PBS, 2 mM ethylenediaminetetraacetic acid (EDTA) and 0.5% bovine serum albumin (BSA) prior to the analysis. After addition of FcR Blocking Reagent (Miltenyi Biotech GmbH, Bergisch Gladbach, Germany) cells were incubated for 10 min at 4 °C in the dark. Cells were then washed again, resuspended in FACS buffer and filtered through a 41 μm mesh. 7AAD was added to the indicated samples. CD133 expression was detected by flow cytometry using the CD133 (293C3)-PE antibody (mouse IgG2b, Miltenyi). Mouse IgG2b-PE isotype control antibodies (Miltenyi) were used to detect non-specific staining and to set gates and marker lines accordingly.

### 2.7. Immunofluorescence Staining, Microscopy and Documentation

Neurospheres were allowed to form by seeding cells in appropriate medium and incubating them for six days. The spheres were then inoculated with amounts of virus corresponding to a multiplicity of infection (MOI) of 0.50 PFU per initially seeded cell. At day three after infection, *i.e.*, day nine of the experiment, cells were transferred to 50 mL tubes, and spheroids were let to settle down by gravity for 20 min. After fixation with 4% paraformaldehyde for 20 min. and settling down, spheroids were washed with PBS, embedded in Tissue-Tek^®^ O.C.T™ cryosection compound (Sakura Finetek Europe B.V., Leiden, The Netherlands) and kept frozen at −20 °C until further use. Spheroids were cut in 8 μm thin cryotome sections. Immunofluorescence detection of the viral protein NS-1 (using the nonstructural protein 1 (NS1) antiserum MK-3) and staining with the dye Hoechst 33342 (SIGMA-Aldrich, St. Louis MO, USA) were performed as previously published. In addition, the expression of the neural stem cell marker Nestin and the astrocytic lineage marker glial fibrillary acidic protein (GFAP) were detected using mouse antibodies specific for human Nestin (hNestin) (R&D Systems, Minneapolis, MN, USA) and anti-GFAP (Progen, Heidelberg, Germany) diluted 1:50 dilution and undiluted, respectively. Secondary staining was done with a 1:700 dilution of Alexa Fluor^®^ 488 (Thermo Fisher Scientific, Waltham, MA, USA). Further steps were performed as described previously [25]. Immunofluorescence was documented by microscopy (magnification: 400×) and digital imaging using a Keyence BZ 9000 microscope. Pictures were taken using the software BZ II Viewer and BZ II Analyzer provided by the manufacturer (version used, KEYENCE Microscope Europe NV/SA, Mechelen, Belgium).

### 2.8. Western Blot Analysis

10,000 cells were either mock-treated or infected with H-1PV at MOIs of 1 or 50 PFU per cell. At time points indicated cells were harvested, pelleted, and washed with cold PBS at 4 °C. Cell pellets were stored at −80 °C until they were lysed for 1 h in radio immunoprecipitation assay (RIPA) buffer on ice. Lysates were centrifuged at 15,000 *g* for 30 min at 4 °C, and protein concentrations in supernatants were determined photometrically using the BIO-RAD protein assay (Bio-Rad GmbH, Munich, Germany) according to the manufacturer’s instructions. 10 μg of protein extracts were fractionated by SDS-10% polyacrylamide gel electrophoresis, and transferred to a polyvinylidene fluoride (PVDF) membrane (Merck Millipore, Billerica, MA, USA).

Mock-infected cultures were used to analyze the neural stem cell markers Nestin (mouse IgG anti human Nestin Clone 196908, R&D Systems; 1:2500) and Sox-2 (mouse IgG anti human/mouse SOX-2 clone 245610, R&D Systems; 1:1000), and the astrocytic lineage marker GFAP (mouse anti-GFAP clone GF 12.24, Progen; dilution 1:200). Polyclonal rabbit antisera (kindly provided by Dr. N. Salomé) were used: for the detection of viral proteins: MK-3 and anti-VP1/2 raised against the viral NS1 and capsid proteins, respectively. Bound primary antibodies were detected using horseradish peroxidase-conjugated IgGs (goat anti-mouse IgG-horseradish peroxidase (HRP) clone sc-2005, and goat anti-rabbit, clone W401B, Santa Cruz Biotechnology, Santa Cruz, CA, USA) in dilutions of 1:2500 or 1:5000, and revealed by chemo-luminescence on X-ray films.

### 2.9. Assessment of Cell Viability and Lysis

Singularized cells were seeded in a 96-well plate (2,500 cells per well) and either infected with iodixanol-purified wild type H-1PV (MOIs of 1, 10, or 50 PFU per cell) or mock-treated with an equal volume of 40% iodixanol (VisipaqueTM, GE Healthcare, Chalfont St Giles, UK) in Ringer solution. The metabolic activity of the cells was quantified using the WST-1 assay according to the manufacturer’s instructions (Roche Diagnostics, Mannheim, Germany). Absorbance of the dye was measured photometrically at 450 nm, with a reference wavelength of 620 nm.

Cell lysis was determined by measuring the release of lactate dehydrogenase into culture medium by use of the Cytotox 96 cytotoxicity assay kit™ (Promega GmbH, Mannheim, Germany). At the time after infection indicated cells underwent triton lysis applying following the manufacturer’s instructions with pelleting the cells at 400 × *g* in v-shaped 96-well plates and transfer of the supernatant necessary for the analysis of neurosphere cultures.

### 2.10. Gene Expression Analyses

Total RNA was collected from the NCH421k cell line and its subclones NCH421I and NCH421R, and processed for microarray analysis using the Affymetrix Human Genome-U133 plus 2.0 array. Sample library preparation, hybridization, and quality control were performed according to the manufacturer’s protocols at the Microarray Department of the University of Amsterdam. Genes were considered to be differentially expressed between the parental cell line (NCH421k) and its parvovirus resistant derivative (NCH421R) when expression was increased or decreased more than 3-fold.

### 2.11. Statistical Analysis

Comparing two means and more than two means was performed using the unpaired student’s *t*-test and ANOVA, respectively. For the comparison of tumor formation frequencies between small groups, the Fisher’s Exact Test were performed using SigmaPlot version 13.0 (Systat Software Inc., San Jose, CA, USA). Copy-number aberrations were determined as described before using the Illumina HumanMethylation450 BeadChip assay [31]. Tumor volume measurements at day 31 were compared with the Mann-Whitney test. One-way ANOVA with Dunnett contrast tests and *p*-value adjustment was used to compare metabolic activity and cytotoxicity between doses or cell lines. In case of variance heterogeneity between groups a robust covariance estimator was used. All tests were two-sided. *p*-values below 0.05 were considered statistically significant. Analyses were performed with software R and add-on package multcomp.

### 2.12. In Vivo Models

7–8 week-old female non-obese diabetic NOD/SCID mice (Janvier Labs, St. Berthevin, France) were kept under pathogen-free conditions. In order to determine, whether H-1PV infection prevented tumor formation, NCH421k cell cultures were either infected with H-1PV at a MOI of one or ten PFU per cell, or mock-treated with serum-free medium. Four hours post-infection, inoculum was removed, 10^6^ CD133 positive NCH421k cells were suspended in PBS-Dulbecco (DPBS, Gibco®, Thermo Fisher Scientific Inc., Waltham, MA, USA) and then injected subcutaneously in to the right flank of the mice (10 animals per group). H-1PV resistant NCH421R cells served as negative controls for the virus treatment and were infected previous to inoculation into the animals as described for NCH421k cells. Tumor growth was monitored three times per week by manual tumor volumetry using an electronic digital caliper (Farnell, Aschheim, Germany). The tumor volume was calculated$\;V=\frac{4}{3}\pi \;a\times \frac{b}{2}\times \frac{c}{2}$ with *b* as length, *c* as width and *a* being the prominence of the tumor according to the ellipsoid model.

## 3. Results

### 3.1. The Neurosphere Cultures Analyzed Represent a Broad Variety of HGG Subtypes But Consistently Display Neural Stem Cell Features

The neurosphere cultures included in the present study originate from six different pediatric and adult grade IV glioma patients aged between three and 67 years (Table 1). Each of the different HGG neurosphere cultures was found to harbor at least one molecular hallmark of HGG. H3.3 mutations were described for the pediatric HGG cultures KNS-42 NS, SU-DIPG IV, and SU-DIPG VI [25,32]. Amplification of *MYCN* (KNS-42 NS) [32] or *MYC* (NCH421k and NCH421R) [33] was observed in one adult and one pediatric GBM neurosphere model. Mutations in *TP53* were previously detected in the pediatric SF-188 adherent culture and parental cell line to SF-188NS [34] and in the adult NCH644 GBM neurosphere culture [33].

**Figure 1:**
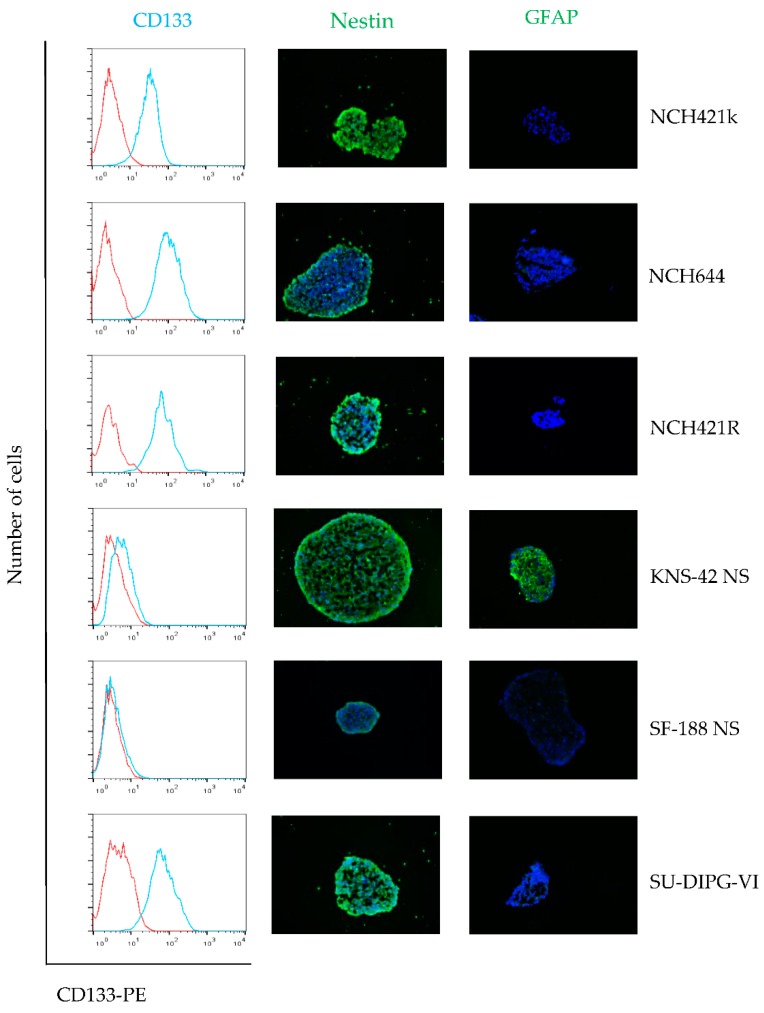
**High-grade glioma (HGG) neurosphere cultures show stem cell features**. Expression of neuroepithelial stem cell marker proteins CD133 and Nestin, and the astrocyte lineage marker glial fibrillary acidic protein (GFAP) in neurosphere cultures were determined by fluorescence-activated cell sorting (FACS) analysis and immunofluorescence microscopy. CD133 expression was assayed by FACS (**cyan**) and isotype control is depicted as the (**red**) respectively. Immunofluorescence for Nestin (**green**) and GFAP (**green**) merged with Hoechst 33342 (**blue**), magnification 400×.

**Figure 2:**
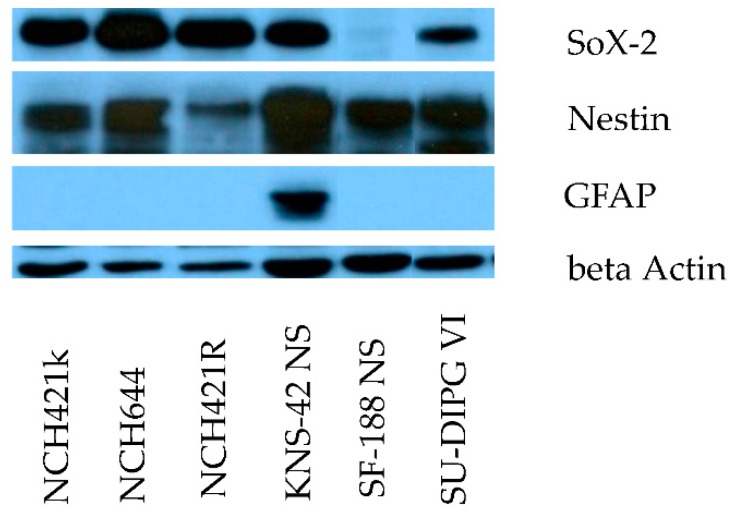
**Stem-like cell expression pattern in HGG neurosphere culture models.** Expression of neuroepithelial stem cell marker proteins SOX-2 and Nestin, and the astrocyte lineage marker GFAP in neurosphere cultures by Western blot. Detection of beta actin served as loading control.

Immunofluorescence and Western blotting analyses showed consistent expression of the neural stem cell and progenitor marker Nestin in the whole panel of neurosphere cultures tested (Figure 1 and Figure 2). In all cultures except for SF-188 NS, a strong expression of the neural stem cell marker SOX-2 could also be demonstrated. In contrast, the early astrocytic lineage marker GFAP was only expressed at a significant level in KNS-42 NS cells, as shown by immune-fluorescence staining and Western blotting (Figure 2). These data are summarized in Table 2 and indicate that all primary neurosphere cultures tested are appropriate models showing a high grade glioma stem-like cell phenotype. In contrast, the expression pattern of stem cell and differentiation markers is only partial in the secondary neurospheres KNS-42 NS and SF-188 NS, showing limitations of this culture approach to model transformed cells at an early step of glial differentiation.

### 3.2. H-1PV Efficiently Transduces and Replicates in Pediatric and Adult HGG Neurosphere Cell Culture Models

**Figure 3:**
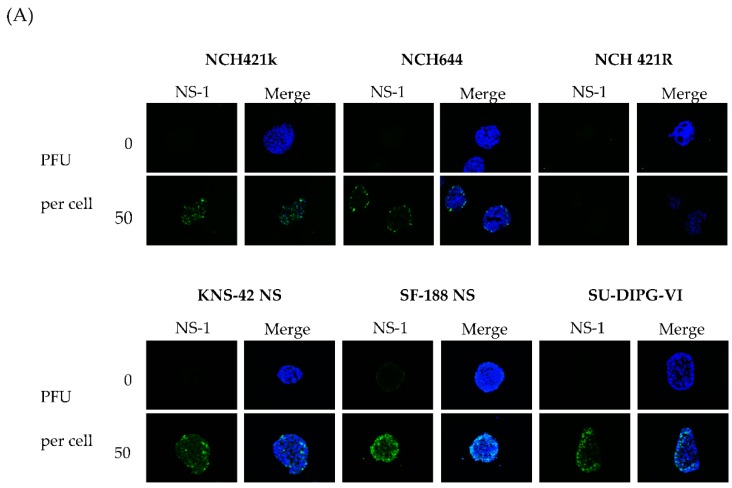

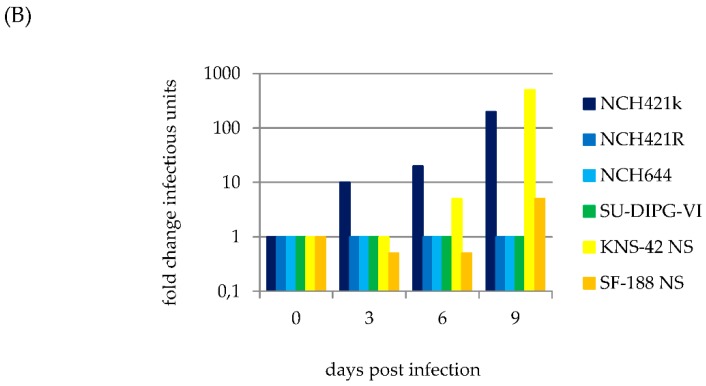
**H-1 parvovirus (H-1PV) initiates replication in adult and pediatric HGG neurospheres.** Indicated HGG neurosphere cultures were infected with H-1PV (one PFU per cell) six days post seeding. (**A**) Three days post infection, the initiation of virus replication was measured by fluorescence microscopy after cell immuno-staining for the nonstructural viral protein 1 (NS1) (**green**), co-staining of neurospheres with Hoechst 33342 (**blue**) is shown in merge panels; (**B**) H-1PV virus production was assayed in a time course experiment by quantification of infectious particles as described in Section 2.5.

All but one neurosphere cultures showed sustained viral protein expression after H-1PV infection, as demonstrated by immune-fluorescence staining for NS1 (Figure 3A). The only exception was the NCH421R subclone which proved to be resistant to parvovirus infection. This subclone derived from NCH421k cells, failed to show any expression of viral proteins, allowing their use as a non-permissive, isogenic HGG neurosphere control in subsequent experiments. Cells were infected with H-1PV at increasing MOIs, and either harvested immediately after virus incubation or at days three, six and nine after infection. An input virus dose as low as one PFU per cell was sufficient to induce significant NS1 and VP1/2 induction. Expression of viral proteins could be detected as early as 72 h after neurosphere infection (Figure 3A) and increased over time (data not shown). Western blot analysis confirmed the expression of proteins NS1, VP1, and VP2 in all susceptible neurosphere cultures in a dose-dependent manner (Figure S2). In all susceptible HGG neurosphere cultures, NS1 and capsid protein expression persisted during the first nine days after infection until the cytotoxic effects of the virus induced complete cell death (see below).

### 3.3. H-1PV Productive Infection is Restricted to a Subset of Pediatric and Adult HGG Neurosphere Cultures

In order to determine whether H-1PV infection of HGG neurosphere cells leads to the production of infectious progeny virions, cultures were infected at an MOI of one PFU per cell and cell culture supernatants were collected at intervals indicated.NCH421R cells, which are unable to express viral proteins upon H-1PV infection (Figure 3A) were used as negative controls and failed to produce infectious viral particles as expected (Figure 3B). Similarly, infection of NCH 644 and SU-DIPG VI neurospheres was non-productive, in spite of the competence of these cells for viral transduction. In contrast, two pediatric (KNS-42 NS, SF-188 NS) and one adult (NCH421k) HGG neurosphere cultures sustained a significant increase in infectious virus titers, demonstrating permissiveness of these cells for progeny virus production. In the cell culture supernatants titers increased from 1,000 IU per mL to 5,000 IU per mL in SF-188 NS, from 5,000 IU per mL to 1,000,000 IU per ml in KNS-42 NS and from 50,000 IU per mL to 10,000,000 IU per mL in NCH421k within nine days after infection with H-1PV at MOI 1 (Figure 3B). The increase in titers of infectious particles ranged from 5-fold in SF-188 NS to 200-fold in KNS-42 NS and NCH421k clearly indicates successful virus replication, assembly and release of progeny virions in these neurosphere cultures.

### 3.4. Infection of Pediatric and Adult HGG with H-1PV Induces Complete Cell Death in All Neurosphere Cultures

**Figure 4:**
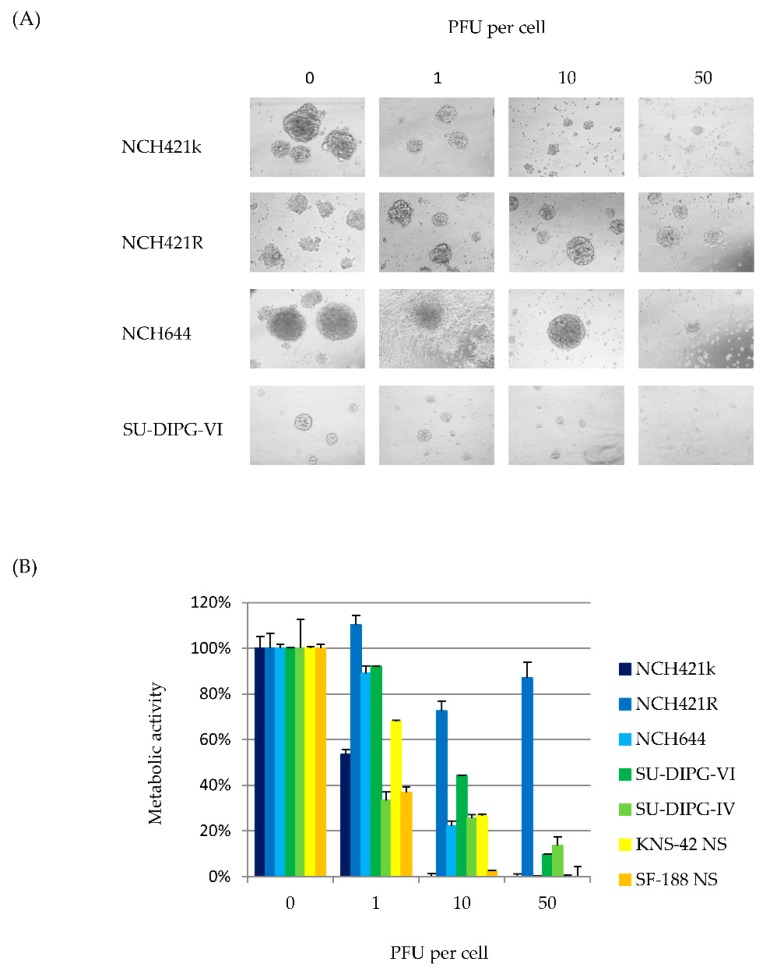
**H-1PV induces cytotoxic effects in HGG “stem-like” cells.** (**A**) Microscopic images of neurosphere cultures infected with indicated doses of H-1PV, nine days post infection, 400× magnification. Neurosphere morphology was determined prior to the toxicity assay; (**B**) At day nine after H-1PV infection at indicated multiplicities, cell metabolic activity was determined by WST-1 assay. Average values with error bars (SEM) from six independent experiments are shown. In all cultures but the resistant subclone NCH421R, 50% inhibition (LD50) was reached after infection with one to ten PFU per cell. After H-1PV infection decrease in metabolic activity in all cell cultures as compared to NCH421R was highly significant (*p* ≤ 0.001).

Cytopathic effects as well cell viability were assayed in the panel of adult and pediatric HGG neurosphere cultures after infection with H-1PV at increasing multiplicities. In neurosphere cultures a significant reduction of the average diameter of neurospheres has to be considered as the equivalent of the well-known cytopathic effects usually observed after H-1PV infection of conventional adherent cell culture models. This cytopathogenic-like effect (CPE-like) was induced in all HGG neurosphere cultures in a viral dose-dependent fashion with the expected exception of the non-permissive NCH421R cells (Figure 4A).

In parallel, H-1PV-induced changes in cell viability were determined by WST-1 assay. Nine days after infection, a striking dose-dependent decrease of metabolic activity was observed in all six permissive HGG neurosphere cultures (Figure 4B). The 50% inhibition (LD50) doses of input virus ranged from one (SU-DIPG-IV, SF-188 NS) to ten (SU-DIPG-VI, KNS-42 NS, NCH421k and NCH644) PFU per cell. Thus, virus-induced cytotoxicity did not significantly differ between pediatric and adult HGG neurosphere cultures. In non-permissive NCH421R cultures, neurosphere size and cell viability remained unaltered after infection with H-1PV at an MOI of up to 50 PFU/cell which led to the complete eradication of susceptible neurosphere cultures. The NCH421R derived subclone therefore represents a model to study H-1PV resistance in HGG neurospheres, as characterized below in further detail.

### 3.5. H-1PV Targets Both CD 133 Positive and CD133 Negative HGG Cells

**Figure 5:**
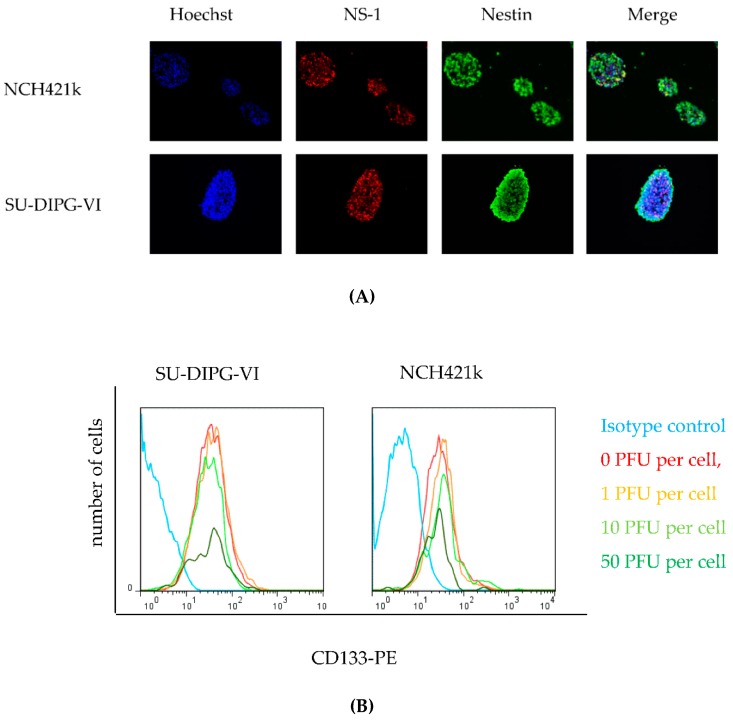

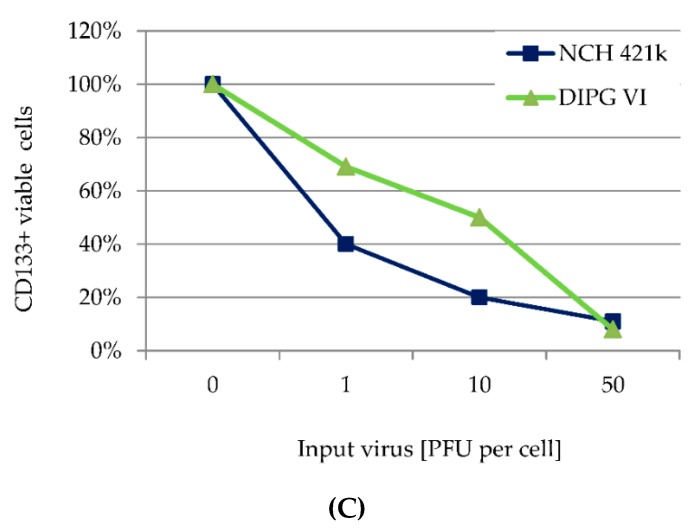
**H-1V infection is cytotoxic to Nestin and CD133 positive cells.** (**A**) Immunofluorescence of two HGG neurosphere cell lines infected with one PFU per cell nine days post seeding, which corresponds to three days post infection. Nuclei of the cells were stained with Hoechst 33342 (**blue**), NS-1 (**red**), Nestin (**green**) and images were merged, 400× magnification. Foci of NS-1 expression colocalize to nuclear staining with Hoechst in Nestin positive cells; (**B**) Cytotoxic effects of H-1PV on CD133-positive cells were assessed by FACS determination of the virus dose-dependent induction of CD133+ cell number at day three after infection. The isotype control was performed using uninfected cells (**cyan**); (**C**) 7AAD negative, *i.e.*, viable cells of two HGG neurosphere cell cultures as determined by FACS nine days post infection with H-1PV at the MOIs indicated.

In order to determine whether H-1PV preferentially killed the stem cell fraction or the differentiating cell subpopulation of neurosphere cultures, the pediatric SU-DIPG-VI and the adult NCH421k neurosphere cultures were analyzed for the expression of stem cell markers after H-1PV infection or mock treatment. For this purpose, cells were incubated with increasing doses of H-1PV at day 6 after establishment of neurospheres. Three days after infection immunofluorescence experiments were performed to detect the viral cytotoxic protein NS-1 (red) and the neuroepithelial stem cell marker protein Nestin (green). Co-staining for both proteins revealed the nuclear accumulation of NS1 in cells showing strong Nestin-positivity and confirmed that H-1PV was indeed able to target stem cells within these neurospheres (Figure 5A). In parallel, the proportion of surviving cells was quantified by 7AAD staining and quantification of living cells (not stained) by flow cytometry. The absolute number of surviving cells decreased with the infectious dose of H-1PV initially administered. In both neurosphere cultures, a similar dose-dependent drop in the survival of CD133+ cells was observed (Figure 5B), showing that the CD133+ and CD133- fraction is target for H-1PV cytotoxic effects. Results were expressed as percentages of living CD133+ cells in virus-infected cultures relative to their mock-infected counterparts (Figure 5C).

### 3.6. H-1PV Resistance Correlates with Dysregulation of Distinct Cellular Genes

**Figure 6:**
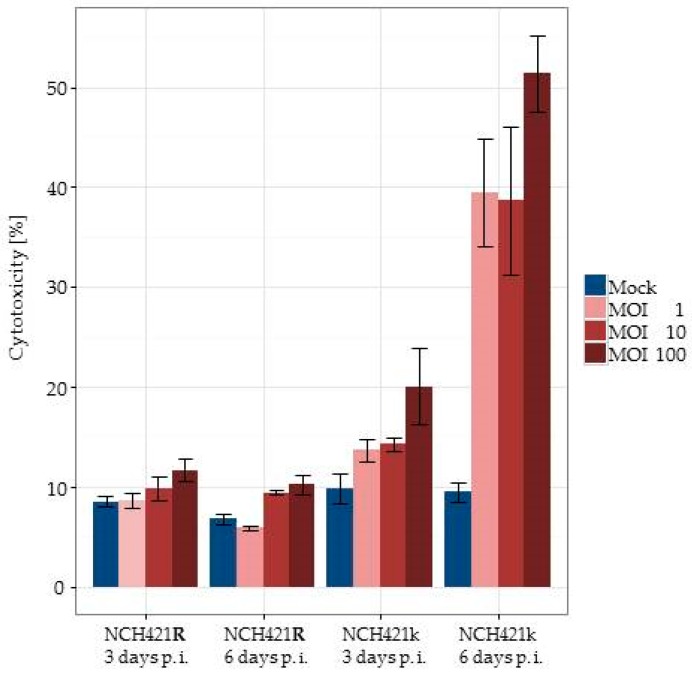
**H-1PV infection induces lytic infection of HGG neurospheres *in vitro*.** Lactate dehydrogenase (LDH)-release assays were performed on NCH421k and NCH421R cells at days 3 and 6 after infection with H-1PV at increasing MOIs. Specific lysis is given in relation to completely lysed cells by detergent treatment. Means from six replicates and respective standard errors are displayed.

Yet NCH421R cells differed from NCH421k cells by the resistance of the former to H-1PV replication and cytopathic effects and the susceptibility of the latter to productive and lytic H-1PV infection (Figure 3, Figure 4 and Figure 6).

**Figure 7:**
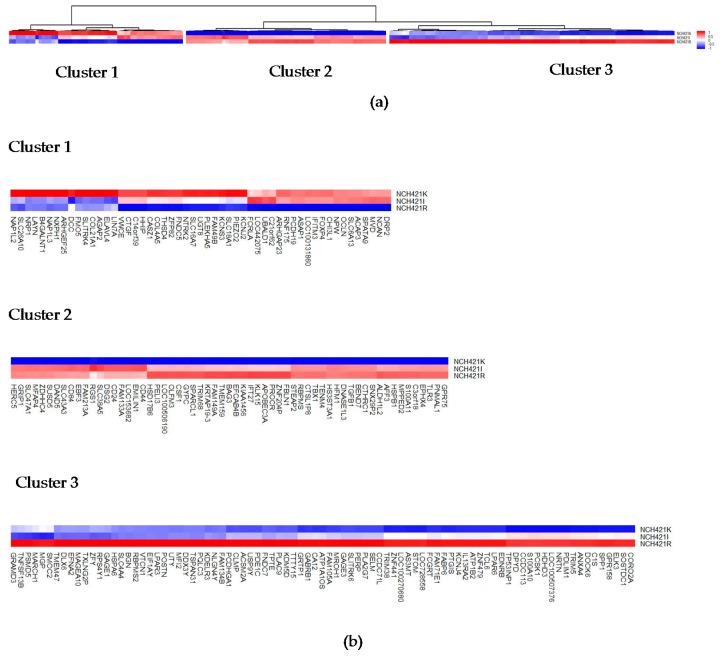
**Comparative transcriptome analysis of the NCH421k and its partially and fully H-1PV resistant subclones NCH421I and NCH421R**. (**a**) Unsupervised clustering of the 201 genes most differentially expressed between NCH421k, NCH421I and the resistant subclone NCH421R. Quantitative comparison of the three profiles reveals genes that are up (**red**) or down (**blue**) regulated in resistant NCH421R cells in comparison with NCH421I and NCH421k cells showing high susceptibility to H-1PV infection; (**b**) Each of the three main clusters is shown independently in order to allow a more detailed analysis.

As depicted in Figure S1 NCH421R cultures could not be distinguished from NCH421k neurospheres regarding their gene copy number profiles, showing both identical HGG hallmark alterations (in particular *MYC* and *PDGFRA* locus amplification). In order to identify candidate genes that may control the susceptibility of HGG stem cells to parvovirus infection transcriptional profiles were compared between the H-1PV-sensitive parental line NCH421k and the resistant subclone NCH421R. This analysis revealed a set of 201 genes that are differentially expressed in the respective cells (Tables S1 and S2). Unsupervised clustering according to the expression patterns in the different clones yielded three different clusters of genes (Figure 7).

Cluster 1 comprises gene hits identified by their reduced gene expression in the H-1PV resistant NCH 421R clone and most significantly over-expressed in the H-1PV susceptible, parental clone NCH421k (*n* = 53), Table S1. These represent putative cellular positive regulators of the parvoviral life cycle. Here *B4GALNT1* and *NTRK2* may be of special interest. *NTRK2* encodes a membrane-bound tyrosine kinase receptor responsible for autocrine growth stimulation of HGG stem cells [36]. Interestingly, another gene hit within this cluster is *IFITM3*, which has been shown to promote GBM cell proliferation and migration [37], which is in good correspondence with the known dependency of parvoviruses on the S-phase of the cell cycle. *B4GALNT1* encodes the protein beta-1,4-*N*-acetyl-galactosaminyl transferase 1 which drives the transfer of acetyl-galactosamine essential for the biosynthesis of the gangliosides GM2 and GD2. These result from sequential glycosylations of glycosphingolipids that contain mono- (M) or di- (D), sialic acid- residues and are active acceptors of sialic acid—a moiety essential for receptor binding by parvoviruses. Since one of the resulting glycophingolipids, GM2, has been shown to act as a co-receptor for reovirus entry [38] into the host cell one may speculate about an analogous role of GM2 or GD2 for the entry of H-1PV.

Genes significantly down-regulated in the H-1PV susceptible parental clone NCH421k and the intermediate responsive clone NCH421I (*n* = 61) were assigned to Cluster 2. One may speculate whether some of these genes encode negative regulators of parvovirus infection. This cluster includes several genes encoding regulators of innate immune response such as *TGFB1* [39], and *TLR3* [40]. It is worth noting that the corresponding proteins are also known or suspected to restrict cell infection with various viruses. The largest gene cluster, Cluster 3, which is characterized by most significant up-regulation exclusively in the resistant subclone NCH421R and may, therefore, encode putative restriction factors for H-1PV infection (*n* = 87). Expression data of genes included in Clusters 2 and 3 are shown in Table S2. Among these additional candidate genes are *APOBEC3A* [40,41], *TRIM5* [42], *CTHRC1* [43], and *TRIM38* [44]. *APOBEC3A*, however, is the innate immune response mediating protein most likely to play a crucial role in H-1PV resistance of HGG cells. This protein has already been described to act as host cell restriction factor for other single-stranded DNA (ssDNA) viruses including the parvoviruses adeno-associated virus serotype 2 (AAV-2) and Minute Virus of Mice (MVM) [41,45]. The role of the other putative candidate genes for host cell restriction factors remains to be identified by further functional studies.

### 3.7. H-1PV Infection of NCH421k HGG Stem-Like Cells Suppresses Tumor Formation in NOD/SCID Mice

To address the clinically relevant question whether H-1PV infection could selectively eliminate HGG stem cells capable of generating HGG tumors *in vivo*, we used a non-obese diabetic (NOD)/SCID subcutaneous xenotransplantation model. The purpose of the experiment presented here, was to show under controlled *in vitro* conditions of infection, that H-1PV is able to abrogate the capacity of CD133 positive glioma stem cells to form tumors *in vivo*. In order to confirm that virus doses below the LD50 were applied, lactate dehydrogenase (LDH) release-assays were performed three and six days after infection with H-1PV at increasing MOIs. Exclusively in NCH421k a dose-dependent glioma stem cell lysis could be observed, which cannot be induced by H-1PV in NCH421R cells (Figure 6). It was of interest to test the effects of H-1PV on the tumorigenicity of the isogenic NCH421R subclone in order to obtain indirect evidence that the suppression of the tumor forming capacity in NCH421k cells is dependent on virus-host cell interactions in course of the H-1PV infection.

**Figure 8:**
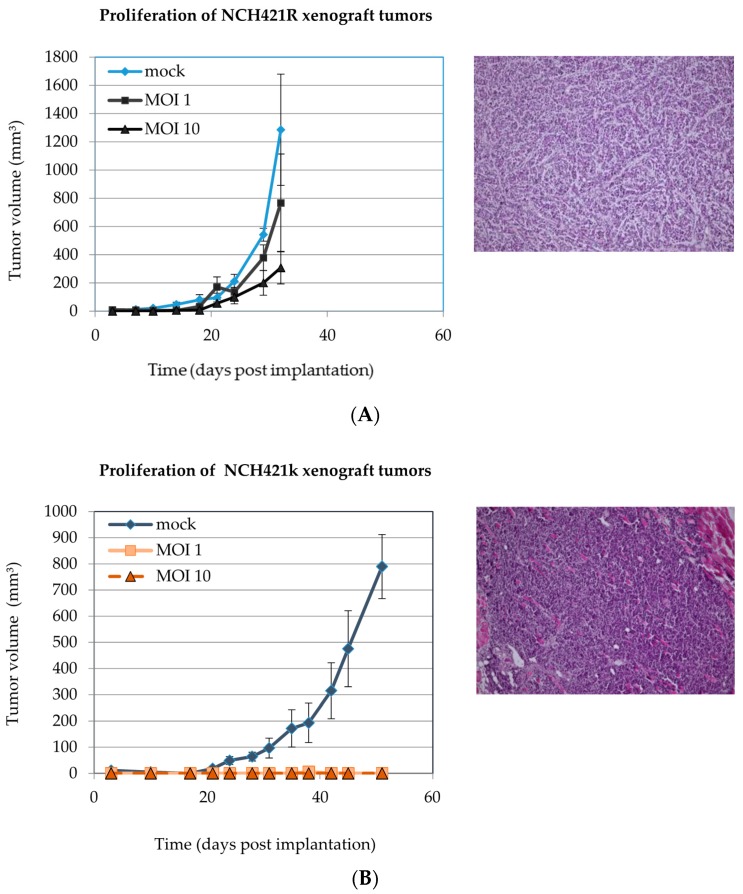
**H-1PV infection suppresses tumorigenicity of HGG neurospheres *in vivo.*** (**A**) NCH421R and (**B**) NCH421k neurosphere cultures were infected with H-1PV (MOI = one or ten PFU/cell) 4 h prior to subcutaneous implantation into the right flank of NOD/SCID mice (10^6^ cells per animal). Mock-treated NCH421k cells served as non-treatment controls. Mock-infected NCH421k and NCH421R xenografts were confirmed to preserve a HGG histomorphology (hematoxylin/eosin, 400× magnification). Significant differences in xenograft tumor proliferation between mock infected cells and H-1PV infected cells were only observed in NCH421k xenograft bearing animals (*p* < 0.001).

Four hours after infection the mock-treated or H-1PV infected cells were implanted into NOD/SCID mice by subcutaneous injection. Implantation of mock-treated NCH421R cells in the NOD/SCID xeno-transplantation model led to the formation of exponentially growing tumors (see Figure 8A) that were undistinguishable from NCH421k tumors with respect to histomorphological patterns (Figure 8B).

All animals inoculated with mock-infected NCH421k cells suffered from the local tumor growth and had to be sacrificed by 40 days after implantation. This was the time point when tumors reached the critical end-point diameter of 30 mm. In contrast, infection with H-1PV suppressed NCH421k cell tumorigenicity in a dose-dependent fashion. Although transient engraftment took place in four out of ten animals treated with MOI 1 and in two out of ten animals treated with MOI 10 H-1PV infection induced delayed tumor regression in these.

At the time point when the animals bearing mock-infected NCH421k xenografts had to be sacrificed, all animals inoculated with H-1PV treated NCH421k cells were tumor free (Table 3). The high grade glioma features of the tumor mock-infected xenografts were confirmed by histological analysis after hematoxylin/eosin staining (Figure 8, right panels). Pre-infection of NCH421R cells with H-1PV at increasing MOIs ranging from one to ten PFU/cell had no significant antiproliferative effect on the development of tumors, showing that the *in vitro* resistance of NCH421R neurospheres to H-1PV infection translated into their *in vivo* resistance to the tumor- suppressing capacity of the virus (see above, Table 3).

## 4. Discussion

Neurosphere cultures are widely accepted as models for pre-clinical drug testing in the HGG stem cell compartment. Adult glioma stem cells share the expression of a panel of proteins physiologically present during early embryonic development of the central nervous system, such as CD133, Nestin, SOX-2 and other less well characterized marker proteins [46]. For pediatric high-grade glioma, no specific “canonical” signature of stem cell markers has been identified so far, which prompted us to characterize the stem cell features of the present panel of HGG neurosphere cultures. In all six pediatric and adult primary neurosphere cultures tested, strong expression of the above mentioned neuroepithelial stem cell marker proteins was demonstrated, indicating their neural stem cell phenotype (Figure 1 and Table 1). However, with loss of SOX-2 expression in SF-188 NS and de novo expression of the glial differentiation marker GFAP in KNS-42 NS, the secondary neurospheres showed some features of differentiation commitment. H-1PV was able to penetrate spheroids and to induce widespread cell infection in all HGG neurosphere cultures—except for the H-1PV resistant subclone NCH421R isolated *in vitro* and used as a negative control throughout this study (Figure 3A).

In some adult and pediatric GBM neurosphere cultures, infection went to completion, leading to the production and release of infectious progeny virions (Figure 3B). These data indicate that, at least to some extent, H-1PV replication takes place in HGG stem cells, and that virus multiplication and spread throughout the tumor may occur in patients. The cellular factors restricting the ability of some HGG cells to support a productive H-1PV infection remain to be identified [47]. It is noteworthy that irrespective of their capacity for sustaining a productive infection, all HGG neurosphere cultures tested were sensitive to the cell-killing effect of H-1PV in a dose-dependent fashion (Figure 4). The sensitivity of HGG stem-like cells to H-1PV toxic activity was independent of the tumor molecular make-up and the patient age or risk profile. The LD50 determined for the adult glioma stem-like cells was in the range of the effective doses previously determined for adherent adult GBM cell lines [48,49]. Interestingly, H-1PV exerted significant cytotoxic effects on both DIPG and GBM neurosphere culture models of pediatric origin at comparably low multiplicities of infection. Co-staining for Nestin and the viral protein NS1 showed that expression of stem cell markers was maintained in HGG cells undergoing viral transduction arguing against a neural differentiation-inducing effect of H-1PV in HGG cells. Accordingly, flow-cytometric analysis of HGG neurosphere cells surviving H-1PV infection showed no enrichment in CD133 negative cells.

By subcloning the NCH421k stem-like cell line, we isolated a spontaneous parvovirus resistant variant that was called NCH421R. NCH421R neurosphere cultures served as negative control for the whole set of experiments described in the present publication. No viral protein production could be detected in these cells after infection with H-1PV. Thus, the resistance of NCH421R cells to H-1PV infection is likely to be due to a block in an early event of the viral life cycle, such as virus entry, nuclear transfer, uncoating, or initiation of viral DNA replication and gene expression. NCH421R cells were indistinguishable from parental NCH421k cells regarding their pattern of genomic alterations and neural stem cell phenotype. The pair of isogenic adult GBM “stem-like” cell neurosphere cultures NCH421k and NCH421R, sensitive and resistant to H-1PV respectively, represents a valuable model to study the molecular mechanisms underlying the susceptibility of HGG stem cells to H-1PV infection.

Among the 201 genes showing highly significant differential expression between NCH421k and NCH421R cells, Cluster 1 comprises genes down-regulated in resistant subclone NCH421R. Remarkably, several of these gene products are known to act as master regulators or key effectors of innate antiviral immune response such as TGFbeta, APOBEC3A, TRIM5, CTHRC1, TRIM38, and TLR3. APOBEC3A is of special interest since this protein, which is induced by innate immune signaling, has been reported to function as host restriction factor for various single-stranded DNA viruses, including the *Parvoviridae* family members AAV2 and MVM. It should be stated, however, that no conclusion can be drawn from this correlation study in the absence of additional groups and functional data, and the present speculations are only made for the sake of further investigations. In conclusion, the transcriptome comparison of H-1PV-resistant versus sensitive isogenic clone of HGG stem cells led us to identify a pool of candidate genes whose involvement in HGG cell permissiveness for parvovirus infection needs to be validated in by functional experiments. This approach is worth being developed further, since the identification of relevant genes may contribute to personalize cancer parvovirotherapy by allowing responsive patients to be selected.

NCH421k neurosphere cultures were previously reported to possess stem-like cell properties and to induce tumors after xenotransplantation of low cell numbers immune-deficient mice [27]. Besides confirming these data, the present study demonstrated NCH421k cell infection with a low H-1PV dose was sufficient to induce full tumor suppression in all mice. The tumor-suppressing effect of H-1PV was dose-dependent, as the fraction of animals failing to show any tumor development increased with the size of the viral inoculum. Therefore, the oncotoxicity of H-1PV for HGG cells *in vitro* translated into efficient antineoplastic activity *in vivo*. As expected, H-1PV failed to prevent virus-resistant NCH421R cells from forming tumors in xenotransplanted mice, even after high multiplicities of infection. Altogether these data show that H-1PV can suppress HGG cell growth under *in vivo* conditions, and confirm the relevance of the NCH421k/R model to investigate determinants of cancer stem cell susceptibility to parvovirus infection.

## 5. Conclusions

The present study demonstrates that the oncolytic parvovirus H-1 can target HGG cells —irrespective of the patients’ age and of tumor histological subtype, of the genetic make-up or the expression pattern for neuronal stem cell markers. In particular DIPG neurosphere culture models are shown for the first time to be sensitive to H-1PV, which has not been reported so far for any oncolytic virus. After infection at sublethal doses, H-1PV was able to prevent adult glioma stem-like cells from subcutaneous engraftment in immunodeficient NOD/SCID mice. Orthotopic transplantation of HGG “stem-like” cells and virus administration through different routes *in vivo* would be required to confirm the antineoplastic efficacy of H-1PV against the corresponding xenograft tumors. This further validation of the use of H-1PV for the elimination of residual glioma cells with stem-like properties is justified by the increasing evidence of the role of these cells in glioma recurrence conferring dismal prognosis.

## Figures and Tables

**Table 1:** Clinical data related to the different tumor tissue donors.

Cell culture	Diagnosis	WHO Grade	Age	Sex	Survival [months]	Ref.
KNS-42 NS	pGBM	IV	16	male	12	[35]
SU-DIPG-IV	DIPG	IV	3	female	6	[25]
SF-188 NS	pGBM	IV	8	male	4,5	[34]
SU-DIPG-VI	DIPG	IV	7	female	8	[25]
NCH644	GBM	IV	67	female	7,5	[33]
NCH421k	GBM	IV	66	male	8,5	[27]
NCH421R	GBM	IV	66	male	8,5	[27]

**Table 2:** Stem cell marker expression and 50% inhibition (LD50) oncolytic parvovirus H-1 (H-1PV) virus doses in high-grade glioma (HGG) neurospheres.

Cell Culture	CD133	Nestin	SOX-2	GFAP	LD50 (PFU per cell)
KNS-42 NS	-	+++	+	+++	1
SU-DIPG-IV	n. d.	n. d.	n. d.	-	1
SF-188 NS	-	++	-	-	10
SU-DIPG-VI	+++	++	++	-	10
NCH644	+++	++	++	-	10
NCH421k	+++	++	++	-	10
NCH421R	+++	+	++	-	>100

GFAP: glial fibrillary acidic protein; PFU: plaque forming units.

**Table 3:** Engraftment of glioma “stem-like” NCH421k cells is prevented by H-1PV infection.

Cell Culture	Virus Dose [PfU per Cell]	Engraftment Rate	
NCH421k	0	10	/10
NCH421k	1	0	/10 *
NCH421k	10	0	/10 °
NCH421R	0	10	/10
NCH421R	1	10	/10
NCH421R	10	9	/10

Equal numbers of NCH421k cells either mock-infected or pre-infected with H-1PV at MOI 1 or MOI 10 were subcutaneously injected into immunodecificent mice. Animals inoculated with pre-infected cells of H-1PV resistant subclone NCH421R served as control groups. * significantly lower engraftment in NCH421k cells (*p* < 0.01) as compared to the NCH421R injected control group treated with H-1PV at the same multiplicity of infection; ° significantly lower engraftment in NCH421k cells (*p* < 0.05) as compared to the NCH421R injected control group treated with H-1PV at the same multiplicity of infection.

## Supplementary Materials

The following are available online at http://www.mdpi.com/1999-4915/8/5/138/s1, **Figure S1**: Copy-number aberrations in NCH421k (A) and the subclones NCH421I (B) and NCH421R (C). **Figure S2**: Expression levels of the viral proteins NS1, VP1 and VP2 in H-1 PV infected neurospheres were measured in HGG neurosphere cultures by Western blot analysis. Cultures were analyzed at indicated days post infection (dpi) with H-1PV at concentrations indicated. **Table S1**: Expression data of 53 genes with significantly lower transcript levels in the partly permissive NCH421I and the completely resistant subclone NCH421R corresponding to Cluster 1. **Table S2**: Expression data of 148 genes with significantly higher transcript levels in the partly permissive NCH421I and the completely resistant subclone NCH421R corresponding to Custer 2 and Cluster 3.
