# Supplementary material for: Pediatric and Adult High-Grade Glioma Stem Cell Culture Models Are Permissive to Lytic Infection with Parvovirus H-1
Source: Viruses. 2016 May 19;8(5):138. doi: 10.3390/v8050138 (PMC4885093; doi:10.3390/v8050138)

Supplementary Materials: Pediatric and Adult High-Grade Glioma Stem Cell Culture Models Are Permissive to Lytic Infection with Parvovirus H-1

Rafael Josupeit, Sebastian Bender, Sonja Kern, Barbara Leuchs, Thomas Hielscher, Christel Herold-Mende, Jörg R. Schlehofer, Christiane Dinsart, Olaf Witt, Jean Rommelaere and Jeannine Lacroix

Table S1. Transcriptome analysis of the uninfected H-1PV permissive NCH421k neurosphere culture and the partially permissive, NCHI and resistant NCHR subclones. Genes most significantly over expressed in NCH421k.

Gene	Probe	NCH421K	NCH421I	NCH421R	R-K
FAM49B	217916_s_at	12.347	8.050	3.536	−8.811
B4GALNT1	1555385_at	11.723	4.907	4.621	−7.102
COL4A5	213110_s_at	6.760	4.000	0.000	−6.760
SLITRK4	232636_at	9.450	2.379	2.744	−6.706
SLC26A10	214951_at	10.967	6.219	4.542	−6.425
IFITM3	212203_x_at	8.086	7.615	2.036	−6.050
ASAP1	224791_at	8.466	8.260	3.000	−5.466
LAYN	228080_at	6.810	1.536	1.379	−5.431
NTRK2	221796_at	8.241	5.741	2.868	−5.373
HHIP	230135_at	5.213	3.838	0.000	−5.213
AGAP2	229917_at	7.181	1.536	2.000	−5.181
PLEKHA5	220952_s_at	6.428	3.982	1.433	−4.995
FNDC5	226096_at	6.000	4.446	1.632	−4.368
ARHGEF25	227427_at	10.759	6.986	6.547	−4.212
CTGF	209101_at	8.195	7.061	4.121	−4.074
CHI3L1	209395_at	5.921	5.517	1.926	−3.995
RNF175	236465_at	6.868	6.287	2.907	−3.961
SLC16A7	207057_at	7.173	5.256	3.293	−3.880
DCC	238914_at	7.668	0.000	3.848	−3.820
SPATA9	223840_s_at	4.062	4.420	0.263	−3.799
LOC100131860	1559180_at	4.372	4.053	0.585	−3.787
C21orf62	220543_at	3.916	5.267	0.138	−3.779
NRP1	212298_at	3.766	0.000	0.000	−3.766
KCNS3	205968_at	7.279	5.194	3.561	−3.718
LIN7A	227929_at	5.336	0.000	1.632	−3.704
ACAP3	225529_at	6.435	6.615	2.744	−3.690
NXPH1	232377_at	10.381	7.070	6.717	−3.664
KCNJ2	206765_at	6.282	4.104	2.678	−3.604
CASZ1	243386_at	4.573	3.154	1.000	−3.573
UBALD1	1558976_x_at	4.329	5.233	0.766	−3.564
THSD4	222835_at	7.941	6.604	4.406	−3.535
VWCE	242957_at	6.418	5.423	2.888	−3.530
C14orf39	1561985_at	3.511	2.561	0.000	−3.511
LOC442075	237005_at	5.267	6.903	1.766	−3.501
FOXP4	227120_at	5.558	5.248	2.138	−3.420
ELAVL4	238073_at	8.095	3.711	4.689	−3.406
DRP2	1556627_at	3.365	3.797	0.000	−3.365
UGT8	228956_at	9.403	7.789	6.070	−3.333
PIEZO2	219602_s_at	6.053	4.027	2.787	−3.267

**ARHGAP23**	**226638_at**	**6.705**	**6.215**	**3.446**	**−3.258**
**SLC18A1**	**207074_s_at**	**5.681**	**3.573**	**2.433**	**−3.248**
**FMO5**	**215300_s_at**	**6.759**	**3.018**	**3.536**	**−3.223**
**NAP1L2**	**219368_at**	**7.101**	**4.542**	**3.897**	**−3.204**
**NAP1L3**	**204749_at**	**8.008**	**5.289**	**4.818**	**−3.191**
**OCLN**	**231022_at**	**6.213**	**6.213**	**3.070**	**−3.143**
**NCAN**	**205143_at**	**10.663**	**11.079**	**7.525**	**−3.138**
**FCRLA**	**235400_at**	**7.348**	**9.076**	**4.263**	**−3.085**
**PCDH19**	**227282_at**	**7.534**	**7.440**	**4.459**	**−3.075**
**ZFP82**	**235176_at**	**6.710**	**5.621**	**3.644**	**−3.066**
**SLC6A13**	**237058_x_at**	**3.322**	**3.433**	**0.263**	**−3.059**
**COL21A1**	**208096_s_at**	**7.168**	**3.828**	**4.129**	**−3.039**
**MVD**	**203027_s_at**	**4.797**	**5.304**	**1.766**	**−3.031**
**NPW**	**243110_x_at**	**8.343**	**8.372**	**5.326**	**−3.018**

Genes discussed in the text are indicated in red.

Table S2. Gene expression analysis of the uninfected H-1PV permissive NCH421k neurosphere culture and the partially permissive, NCHI and resistant NCHR subclones. Genes most significantly over expressed in NCH421R.

Gene	Probe	NCH421K *	NCH421I	NCH421R *	R/-K *
CTHRC1	225681_at	1.632	11.146	11.739	10.107
**RPS4Y1**	201909_at	3.755	2.036	12.212	8.457
**EIF1AY**	204409_s_at	1.722	1.807	9.589	7.867
**USP9Y**	228492_at	0.379	2.170	7.877	7.498
**DDX3Y**	205000_at	3.087	3.278	10.350	7.263
**DPYD**	204646_at	1.379	5.752	8.519	7.140
**LPAR3**	231192_at	0.000	0.000	7.081	7.081
**UTY**	208067_x_at	0.766	1.000	7.489	6.724
**PNMAL1**	218824_at	3.263	9.139	9.951	6.688
**LPAR6**	218589_at	0.000	2.963	6.594	6.594
**S100A10**	200872_at	2.322	6.562	8.908	6.586
**BEND7**	227341_at	0.000	6.096	6.438	6.438
**GAGE3**	207663_x_at	1.070	2.848	7.402	6.331
**FNDC7**	240837_at	1.322	2.233	7.194	5.872
**MAGEA10**	210295_at	0.926	0.138	6.796	5.870
**CA12**	214164_x_at	2.828	5.070	8.663	5.835
**STEAP2**	225871_at	1.632	6.966	7.384	5.751
**TP53INP1**	225912_at	0.000	3.472	5.618	5.618
**POSTN**	210809_s_at	0.263	0.263	5.860	5.597
**NLGN4Y**	207703_at	2.828	3.170	8.392	5.564
**SELM**	226051_at	0.000	1.632	5.495	5.495
**RBPMS2**	228802_at	0.138	0.000	5.618	5.480
**HS3ST3A1**	219985_at	0.000	5.365	5.426	5.426
**TXLNG2P**	236694_at	2.888	2.202	8.176	5.288
**FAM149A**	214889_at	0.000	3.818	5.282	5.282
**GRTP1**	229377_at	0.138	2.070	5.361	5.224
**TRIM38**	203568_s_at	1.138	3.689	6.311	5.174
**ZDHHC4**	220261_s_at	1.379	6.815	6.550	5.171
**KDM5D**	206700_s_at	2.138	3.138	7.296	5.158
**NRTN**	210683_at	0.138	3.350	5.282	5.144
**SPP1**	209875_s_at	7.371	10.076	12.441	5.071
**FAM133A**	231131_at	0.926	7.261	5.993	5.067

**KDELR3**	204017_at	5.340	5.845	10.388	5.048
**TCL6**	219840_s_at	0.000	2.170	4.863	4.863
**EFCAB4B**	228752_at	1.202	4.678	6.057	4.856
**KIAA1456**	239297_at	1.000	4.459	5.820	4.820
**ZFY**	230760_at	2.485	1.322	7.282	4.796
**KCNJ4**	208359_s_at	1.926	4.027	6.688	4.762
**ZNF441**	1553193_at	0.000	2.293	4.684	4.684
**TGFB1**	203085_s_at	0.000	4.365	4.667	4.667
**S100A11**	200660_at	0.926	4.892	5.533	4.607
**GAGE1**	208283_at	1.585	0.678	6.162	4.577
**PELI3**	235431_s_at	1.485	4.888	5.993	4.508
**HSD17B6**	205700_at	2.585	5.888	7.003	4.418
**SPARCL1**	200795_at	8.284	11.656	12.655	4.371
**BGN**	213905_x_at	1.766	1.632	6.094	4.328
**EPHX4**	239579_at	2.848	6.684	7.171	4.323
**AFF3**	227198_at	2.263	5.805	6.567	4.304
**SOSTDC1**	213456_at	2.459	4.776	6.713	4.253
**EFNA2**	238956_at	1.848	1.263	6.036	4.188
**SLC47A1**	219525_at	3.632	8.086	7.806	4.174
**DOCK6**	221794_at	1.322	3.733	5.485	4.163
**HDHD3**	221256_s_at	1.202	3.766	5.274	4.073
**KRTAP19-3**	240967_at	0.585	3.511	4.615	4.030
**GRAMD3**	218706_s_at	1.585	0.000	5.615	4.030
**CORO2A**	227177_at	1.585	3.776	5.591	4.006
**TBX1**	236926_at	2.848	6.451	6.777	3.929
**HFM1**	241469_at	0.000	3.828	3.907	3.907
**APOBEC3A**	210873_x_at	0.000	2.700	3.888	3.888
**KLK15**	221462_x_at	0.138	2.828	4.018	3.880
**C3orf18**	219114_at	1.202	4.498	5.040	3.838
**IL13RA2**	206172_at	0.000	1.678	3.818	3.818
**TENM4**	213273_at	2.722	6.198	6.531	3.809
**PROCR**	203650_at	4.365	7.007	8.170	3.805
**GYPC**	202947_s_at	3.561	6.492	7.349	3.788
**MPPED2**	205413_at	4.973	8.243	8.760	3.788
**MFAP4**	212713_at	2.848	6.914	6.634	3.786
**FABP6**	210445_at	4.706	6.489	8.487	3.781
**TRIM5**	210705_s_at	3.472	5.703	7.252	3.779
**FAM105A**	219694_at	2.170	3.365	5.897	3.727
**LOC100270680**	1561232_at	2.744	4.561	6.471	3.727
**PCSK1**	205825_at	1.485	3.868	5.198	3.712
**CCDC71L**	226756_at	4.365	5.443	8.075	3.710
**SLITRK6**	232481_s_at	4.154	5.202	7.857	3.703
**SNX29P2**	214712_at	0.263	3.755	3.963	3.700
**HERC5**	219863_at	3.121	6.977	6.819	3.698
**PCDHGA1**	211880_x_at	0.379	1.322	4.070	3.692
**EDNRB**	204273_at	5.840	7.492	9.522	3.681
**EBF3**	227242_s_at	0.000	4.225	3.678	3.678
**SUSD5**	214954_at	5.443	9.332	9.117	3.674
**GRIP1**	235957_at	2.536	6.320	6.209	3.673
**RBPMS**	209487_at	2.585	5.970	6.248	3.663
**EMILIN1**	204163_at	1.070	5.773	4.706	3.636
**MFI2**	235911_at	2.293	2.433	5.916	3.624

**PLA2G7**	206214_at	2.070	3.170	5.684	3.613
**CD84**	211189_x_at	0.379	4.661	3.991	3.612
**GPR158**	232195_at	2.406	4.285	5.977	3.571
**FCGRT**	218831_s_at	3.963	5.453	7.534	3.570
**ELK3**	221773_at	5.372	7.347	8.917	3.545
**CD24**	216379_x_at	5.567	9.992	9.102	3.535
**C1S**	208747_s_at	1.000	3.018	4.524	3.524
**PERP**	222392_x_at	6.211	7.214	9.730	3.519
**CTSL1P8**	1563445_x_at	0.766	3.926	4.271	3.505
**CLMP**	228082_at	1.202	2.000	4.700	3.499
**DAND5**	1562772_a_at	0.766	4.615	4.233	3.467
**DNASE1L3**	205554_s_at	0.138	3.446	3.585	3.447
**HSPA6**	213418_at	4.945	4.744	8.388	3.443
**TMEM47**	209656_s_at	0.379	0.000	3.818	3.439
**ATP1A1OS**	236623_at	1.138	2.433	4.573	3.435
**PDLIM1**	208690_s_at	3.700	5.855	7.135	3.435
**SLC38A5**	234973_at	3.036	7.918	6.461	3.425
**ZNF204P**	214823_at	0.848	3.263	4.271	3.423
**PDE1C**	236344_at	4.113	4.926	7.524	3.411
**ANXA4**	201301_s_at	5.198	7.212	8.597	3.400
**TMEM159**	213272_s_at	3.722	6.178	7.122	3.400
**OLFM3**	1554526_at	2.104	4.760	5.495	3.391
**TPTE**	220205_at	0.138	0.678	3.524	3.386
**TLR3**	206271_at	1.379	4.385	4.760	3.382
**PTGIS**	208131_s_at	3.878	5.443	7.248	3.370
**ATP1B2**	204311_at	3.170	4.644	6.525	3.355
**TNFSF13B**	223502_s_at	3.121	1.536	6.472	3.351
**FAM71E1**	229289_at	1.766	3.170	5.113	3.347
**LOC100507376**	229189_s_at	1.963	4.070	5.307	3.344
**MGP**	202291_s_at	2.982	0.000	6.318	3.336
**LOC153682**	232794_at	0.000	4.170	3.336	3.336
**AS3MT**	223652_at	2.104	3.776	5.436	3.332
**ZNF479**	1555367_at	1.202	2.655	4.511	3.309
**LOC100506190**	217625_x_at	2.104	4.597	5.406	3.302
**TTTY15**	214983_at	2.868	3.524	6.154	3.286
**FAM134B**	218532_s_at	5.389	5.555	8.662	3.273
**CCDC113**	222890_at	3.170	5.166	6.440	3.270
**PLAC9**	227419_x_at	0.379	1.070	3.644	3.265
**PSMD5**	203447_at	2.963	1.263	6.227	3.263
**FBLN1**	202994_s_at	3.379	5.667	6.641	3.262
**TSPAN31**	203227_s_at	9.000	9.066	12.243	3.243
**HSPB1**	201841_s_at	5.263	7.940	8.495	3.232
**BAG3**	217911_s_at	5.517	7.811	8.734	3.216
**GPR75**	220481_at	0.926	3.733	4.129	3.203
**SLC4A4**	203908_at	4.591	4.548	7.765	3.174
**MROH1**	1565653_at	0.000	1.000	3.170	3.170
**ACSM2A**	244723_at	1.848	2.609	5.005	3.157
**VTCN1**	219768_at	1.000	0.926	4.146	3.146
**SMOC2**	223235_s_at	2.632	0.138	5.768	3.136
**MARCH1**	235385_at	3.233	1.138	6.368	3.135
**TRIM68**	219405_at	1.722	4.129	4.843	3.121
**SLC43A3**	213113_s_at	4.379	7.892	7.497	3.119

**STOM**	201061_s_at	6.170	7.756	9.286	3.116
**DLX6**	242940_x_at	4.954	4.485	8.053	3.098
**RGS1**	216834_at	0.379	5.687	3.472	3.094
**IFT27**	205037_at	2.926	5.022	6.018	3.092
**PQLC3**	225579_at	3.498	3.561	6.588	3.090
**DSG2**	217901_at	5.731	9.590	8.793	3.062
**LOC728558**	1560352_at	1.322	2.888	4.379	3.057
**CD44**	212063_at	3.087	7.143	6.138	3.050
**GABRB1**	207010_at	3.186	4.293	6.208	3.022
**CSF1**	209716_at	3.053	5.358	6.075	3.022
**FAM213A**	228155_at	7.357	10.850	10.372	3.015
**ALDH1L2**	231202_at	4.162	6.583	7.171	3.009

Genes discussed in the text are indicated in red.

Figure S1. Copy-number aberrations in the three NCH421 clones. Data presented for NCH421k (A) and the subclones NCH421I (B) and NCH421**R** (C) were obtained based on the Illumina HumanMethylation450 BeadChip assay.

(A)

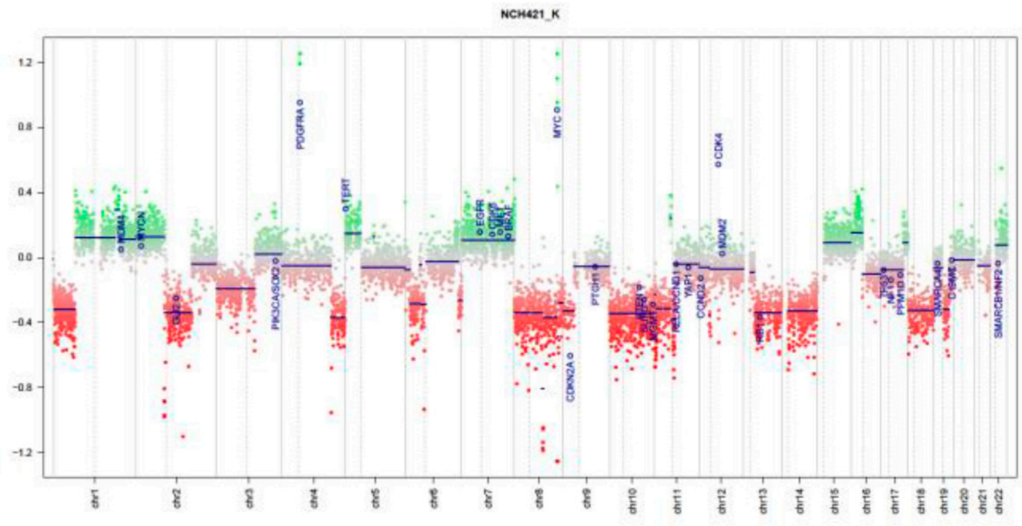

(B)

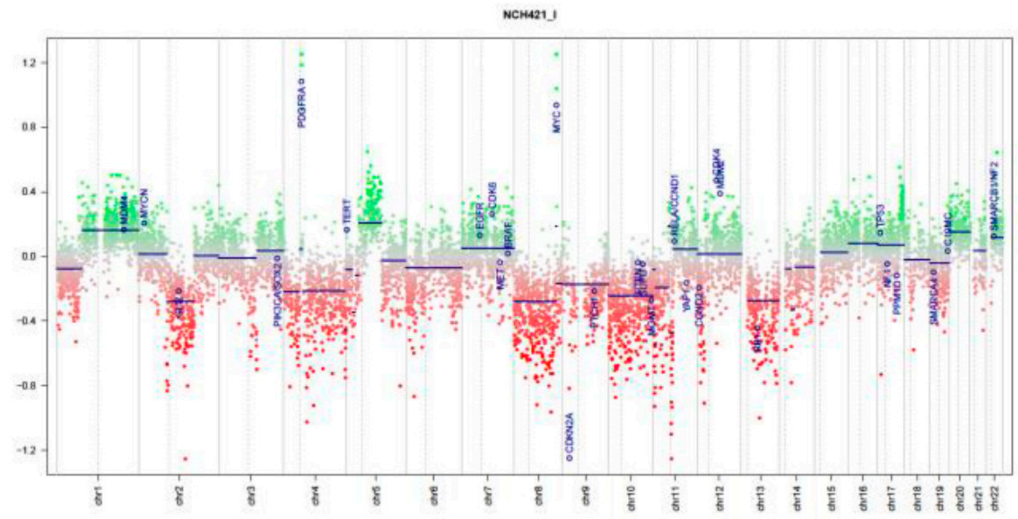

(C)

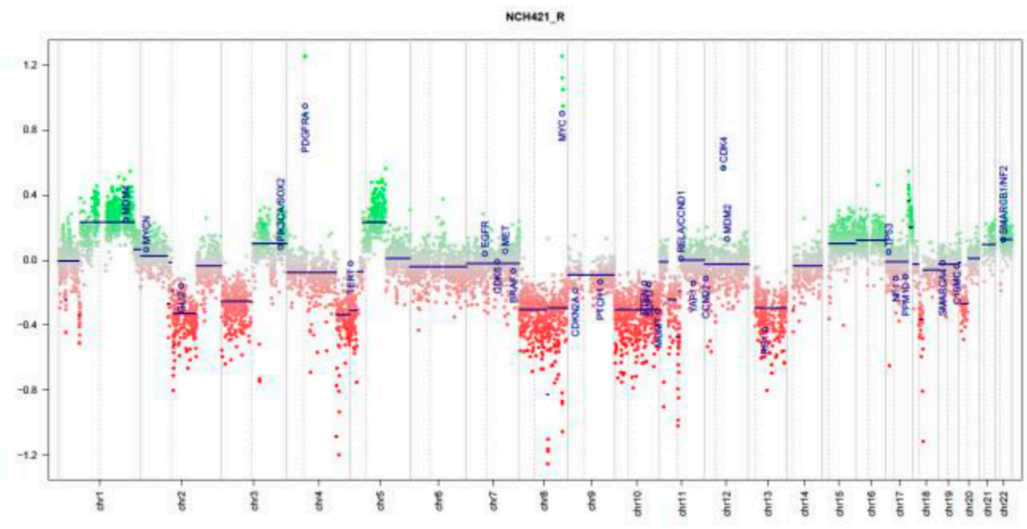

Figure S2. Expression levels of the viral proteins. NS1, VP1 and VP2 in H-1 PV infected neurospheres were measured in HGG neurosphere cultures by Western blot analysis. Cultures were analyzed at indicated days post infection (dpi) with H-1PV at concentrations indicated.

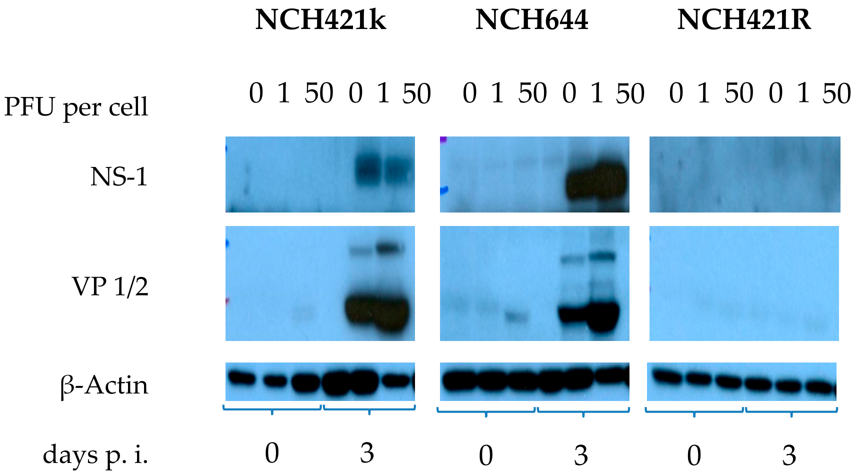

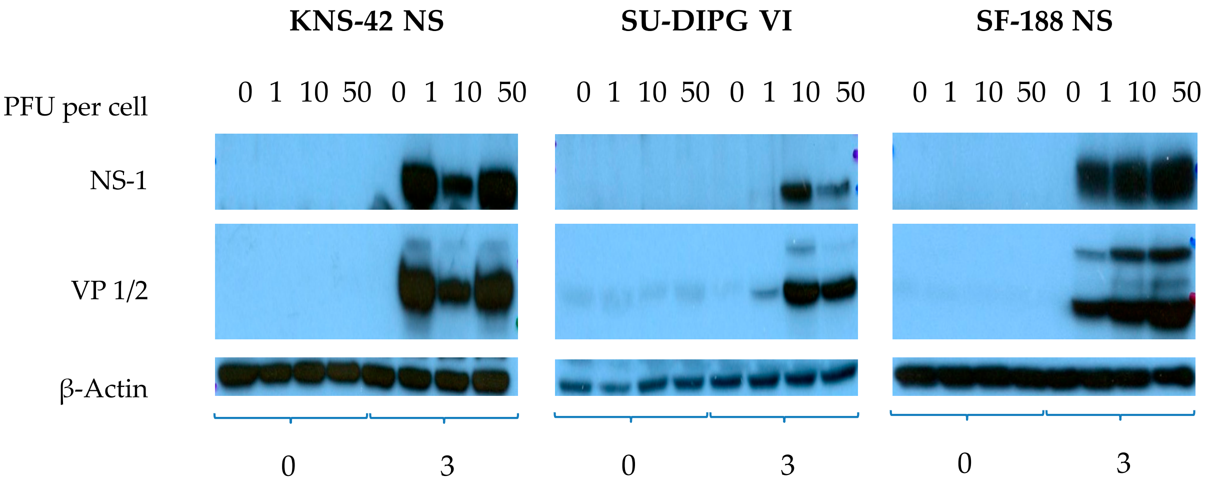

Supplement: Supplementary file 1 [file viruses-08-00138-s001.pdf]
